# Experimental and Numerical Investigation of the Interfacial Bonding in Multi-Material Fused-Filament Fabrication Using Mode-I Fracture Toughness

**DOI:** 10.3390/polym18202529

**Published:** 2026-10-15

**Authors:** Ermias Wubete Fenta, Mohd Tauheed, Sarfaraz Ahmed Furquan, Ammar Alsheghri

**Affiliations:** 1 Department of Mechanical Engineering, King Fahd University of Petroleum and Minerals (KFUPM), Dhahran 31261, Saudi Arabia; 2 Interdisciplinary Research Center for Biosystems and Machines, King Fahd University of Petroleum and Minerals (KFUPM), Dhahran 31261, Saudi Arabia; mohd.tauheed@kfupm.edu.sa

**Keywords:** multi-material additive manufacturing (MMAM), fused-filament fabrication (FFF), Mode-I fracture toughness, cohesive zone modeling (CZM), raster orientation

## Abstract

Multi-material fused-filament fabrication (FFF) enables the integration of two or more materials within a single printed component, offering significant potential for producing functionally graded and hybrid structures with tailored mechanical properties. However, the effects of processing conditions on interfacial quality and fracture behavior in multi-material FFF remain insufficiently understood. This study investigates the Mode-I interfacial fracture behavior of multi-material FFF using compact-tension (CT) specimens through combined experimental testing and finite-element simulation. CT specimens were fabricated from polylactic acid (PLA), thermoplastic polyurethane (TPU), and polyethylene terephthalate glycol (PETG) using five raster configurations, including rectilinear 0°/90°, ±45° and aligned-rectilinear 0°, 45°, 90° configurations. The load-extension response, peak load (P_max_), critical stress-intensity factor (K*_IC_*), and critical strain-energy release rate (G*_IC_*) were evaluated. Two-way ANOVA showed that material pairing was the dominant statistically significant factor affecting peak load, whereas raster orientation and its interaction with material pairing were not statistically significant. The PETG–TPU interface exhibited the highest fracture resistance, with the AR-45 orientation producing an average P_max_ of 130.90 N, K*_IC_* of 0.761 $\mathrm{MPa}\sqrt{m}$, and G*_IC_* of 375.5 N/m. Fracture-surface observations supported these findings, revealing greater deformation, material transfer, surface roughness, and mechanical interlocking in the PETG–TPU specimens. The calibrated cohesive-zone model demonstrated the stress contours and the localized interfacial damage patterns. Overall, material pairing primarily governed interfacial fracture resistance, whereas raster orientation showed no statistically significant effect under the tested conditions.

## 1. Introduction

Additive manufacturing (AM) has emerged as an important manufacturing technology for producing geometrically complex and customized components with reduced material waste and increased design flexibility [1,2]. Recent advances in AM include multi-material additive manufacturing (MMAM) wherein two or more materials with varied physical and mechanical properties can be incorporated into one component. This process enables the fabrication of functionally graded and hybrid materials in which variations in the functional properties like stiffness, flexibility, strength, thermal characteristics, etc., can be created [2,3]. Moreover, incorporation of dissimilar materials during the process avoids secondary processes of assembly such as bonding and welding. Among the polymer-based AM methods, the fused-filament fabrication (FFF), also known as the fused deposition modeling (FDM) technique, offers great potential for multi-material AM. It offers low cost, feasibility, and material flexibility, and the possibility of depositing various thermoplastic filaments using multiple extruders [4,5]. In the case of FFF, the thermoplastic filament is heated above its softening/melting temperature and then extruded via a nozzle on top of pre-programmed paths to create a component in a layer-by-layer fashion. With the advent of the multi-nozzle printers, this potential has been greatly increased by being able to use different polymers during the deposition of the same component, even in some cases, the same layer [6,7]. Thus, multi-material FFF offers considerable possibilities for production of rigid–flexible structures, functionally graded parts, light-weight structures, biomedical devices, and mechanically engineered parts.

Despite these advantages, the interface between dissimilar materials is a crucial factor in determining the structural properties and reliability of multi-material FFF structures. In contrast to single-material printing, multi-material interfaces may include polymers that differ significantly from each other in their melting temperatures, viscosities, coefficients of thermal expansion, shrinkage, stiffness, and molecular composition. This may limit molecular diffusion between the materials, create residual stress during cooling, and reduce the bonding strength between adjacent materials [4,5,8]. The deposition process ensures that the material is kept hot enough for bonding to occur. For incompatible polymers, it is possible that bonding will not take place by diffusion alone [9]. Chemical incompatibility may limit intermolecular interactions, leaving the mechanical strength of the interface to be determined by molecular adhesion and geometrical/mechanical interlocking formed during the printing process. Consequently, the failure of a multi-material printed structure may begin at the interface rather than at a certain constituent material despite the relatively high mechanical strength of individual materials [10,11].

A wide range of thermoplastic materials is currently available for FFF, including polylactic acid (PLA), thermoplastic polyurethane (TPU), polyethylene terephthalate glycol (PETG), acrylonitrile butadiene styrene (ABS), polypropylene (PP), polyethylene (PE), and polyamide (PA) [12,13]. Various material combinations have been investigated, such as PLA/ABS, PLA/TPU, conductive polylactic acid (cPLA)/ABS, PLA/TPU, chlorinated polyethylene (CPE)/TPU, CPE/PLA, PLA/conductive-PLA, ABS/conductive PLA, and ABS/TPU, etc. [6,14,15,16,17,18]. From the materials mentioned above, PLA, TPU, and PETG seem to be the most exciting for multi-material fabrication because of their differing mechanical and thermal properties. While PLA demonstrates relatively high stiffness and printability, TPU is characterized by relatively high flexibility and the ability to undergo large deformations. PETG has relatively good strength, toughness, resistance to chemicals, and dimension stability. Thus, incorporation of these polymers into a single structure allows one to manufacture components with different stiffness, flexibility, and robust zones [13,19,20]. However, there are some difficulties associated with these differences in the properties of the materials as they create challenges related to interfacial bonding quality between these materials.

The mechanical performance of multi-material interfaces depends not only on the choice of materials used, but also on processing and printing parameters. The interface formed at the geometric boundary between dissimilar materials is regarded as the critical element in MMAM components, which varies with the properties and printing conditions of the materials [11,21]. Parameters such as nozzle temperature, deposition speed, layer thickness, direction of the raster, and pattern of infill have an impact on thermal profile, area of the interface, polymer chain diffusion, and stress distribution in the interface [22,23]. Among these parameters, printing orientation of filaments gives rise to anisotropic behavior of FFF printed parts, as deposition of roads leads to preferential directions of load transfer and crack propagation. Orientation of the raster in relation to an existing crack and loading direction may affect the initiation of the crack, crack deflection, separation between roads, and failure of the interface [24].

Various experiments have been carried out to investigate the nature of bonding between different types of printed polymers. These include tensile, lap-shear, peel, and fracture tests [25,26]. While standard strength testing provides important information about the load at which failure occurs at an interface, there are more accurate methods based on fracture mechanics that can evaluate the resistance of an interface to crack initiation and growth [27]. Specifically, Mode-I loading is a loading type where the fracture is dominated by opening of the interface and can be used to evaluate the sensitivity of an interface to delamination. A compact-tension specimen is widely used to evaluate fracture behavior because the stress field is localized around a pre-crack. Thus, it is possible to calculate the value of the critical stress intensity factor (fracture toughness (K_IC_)) and critical strain energy release rate (G_IC_) using the critical maximum load (P_max_) and specimen dimensions.

However, the major limitation of the existing body of literature lies in the fact that the Mode-I behavior of the interfaces of dissimilar multi-material FFF assemblies is inadequately studied. Most prior research efforts were based on tensile, lap-shear, or peel/shear tests, which is inadequate in terms of studying crack initiation and propagation [28,29]. Even though some research has been done regarding the interfacial bonding and Mode-I fracture properties of dissimilar material fabricated using multi-material FFF for certain combinations of materials like PLA/Nylon, and CFPA/TPU, the majority of fracture experiments have been carried out using Double Cantilever Beam (DCB) test or similar tests [28]. Consequently, experimental investigations using Compact Tension (CT) specimens for these multi-material interfaces remain limited. There is a gap in the determination of Mode-I fracture properties of multi-material FFF using the CT test. Moreover, there are relatively few studies that combine experimental Mode-I fracture testing with numerical simulations. Numerical simulations, especially the cohesive zone modeling (CZM), have not been properly validated through experimental studies of the fracture of the multi-material FFF interface. Few efforts have been made to incorporate such characterization using CT-based experiments in combination with validated numerical modeling of crack initiation, propagation, and growth across dissimilar-material interfaces. Moreover, only limited studies have combined experimental testing with numerical modeling of crack initiation at dissimilar-material interfaces. In addition, while the effect of raster orientation and material type on the Mode-I fracture toughness for single-material FFF parts was investigated [30], their influence on the interfacial K_IC_ and G_IC_ of dissimilar multi-material FFF specimens through CT specimens has not been sufficiently explored. The novelty of this research lies in the development of an integrated experimental-numerical CT approach for evaluating Mode-I interfacial fracture properties and fracture initiation behavior in dissimilar material FFF interfaces.

This study experimentally and numerically investigates the Mode-I fracture behavior of multi-material FFF structures fabricated from PLA, TPU, and PETG. The compact tension (CT) specimens incorporating PLA–PETG, PLA–TPU, and PETG–TPU interfaces were tested to determine the peak load (P_max_), critical stress intensity factor (K_IC_), and critical-strain energy release rate (G_IC_). The effect of raster orientation was examined using aligned-rectilinear patterns of 0°, 45°, and 90°, together with alternating rectilinear patterns of 0°/90° and +45°/−45°. The specimens were fabricated using Bambu Lab H2D FFF printer, and the experimental investigation was complemented by finite-element analysis. A cohesive-zone model (CZM) was employed to represent interfacial crack initiation and damage evolution in the multi-material FFF specimens. The combined experimental and numerical approach was used to evaluate the effect of material pairing, examine the response of the different raster configurations, and determine whether raster orientation produced a statistically significant effect on interfacial fracture behavior.

## 2. Materials and Methods

### 2.1. Materials

In this study, three commercially available thermoplastic filaments, PLA, TPU, and PETG filaments were utilized, to investigate the interfacial bonding and Mode-I fracture behavior of multi-material specimens fabricated by FFF. All filaments were supplied by Bambu Lab (Shenzhen, China) with a nominal diameter of 1.75 mm. Specifically, Bambu Lab PLA Basic, TPU for AMS, and PETG Translucent were employed in this study. The chosen polymers possess different mechanical and thermal properties that make them suitable for investigating the effect of material incompatibility on the behavior of interfacial fracture. PLA is relatively rigid and thermoplastic, while TPU is more flexible and deformable. PETG has an intermediate set of mechanical and thermal properties. In this way, the choice of these three materials makes it possible to create interfaces between polymers with different mechanical and thermal properties. This selection of materials facilitates a focused assessment of the interfacial adhesion challenges between a rigid filament and flexible counterpart, contributing to the broader understanding of multi-material 3D printing dynamics.

### 2.2. Specimen Design and Fabrication

**Figure 1:**
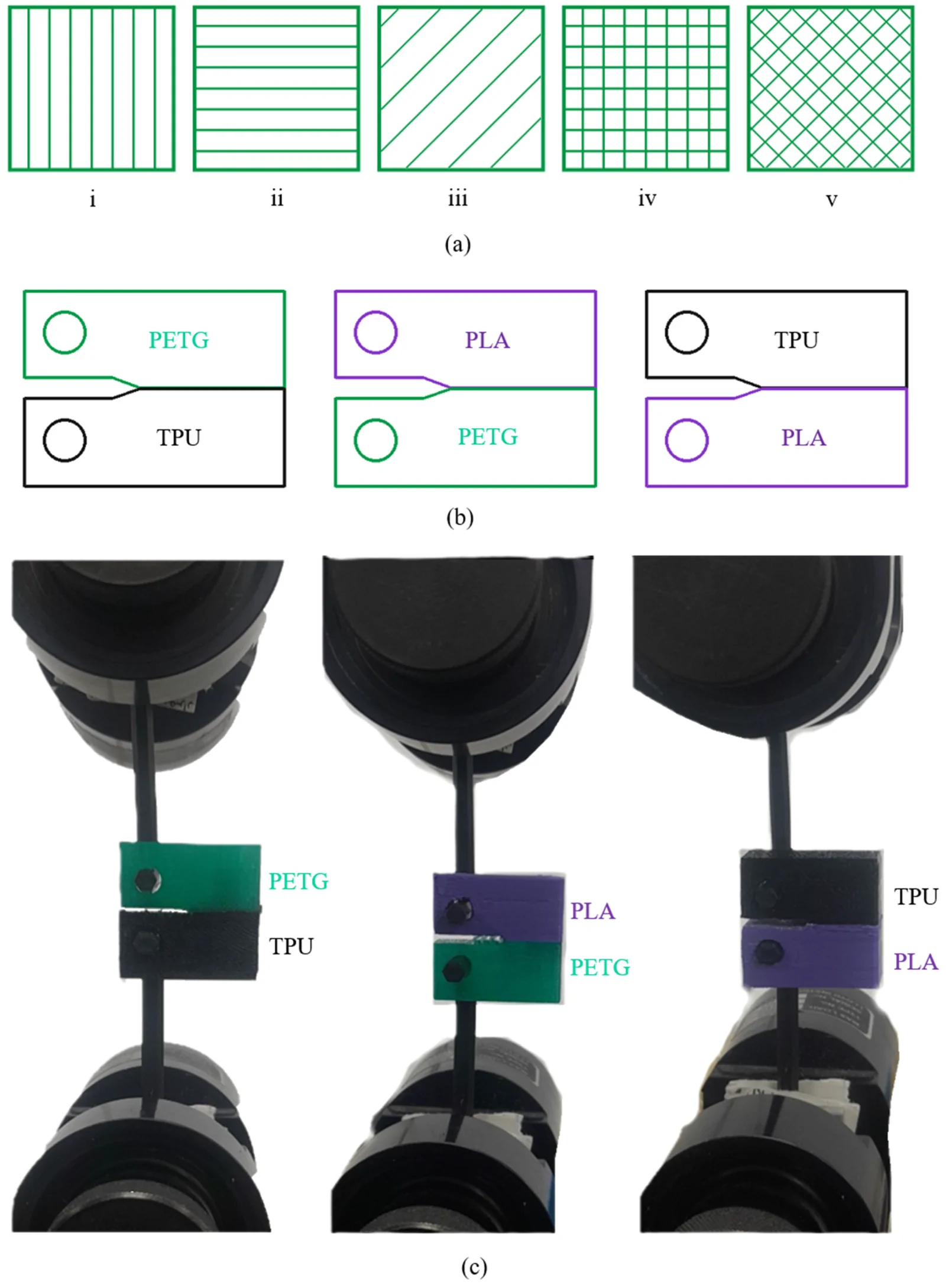
(**a**) Raster orientation: (i) aligned rectilinear 0°; (ii) aligned rectilinear +45°; (iii) aligned rectilinear 90°; (iv) rectilinear 0°/90°; (v) rectilinear +45°/−45°. (**b**) ASTM E399 CT specimen geometry with three dissimilar material combinations model for printing. (**c**) CT test setup.

The CT specimens were used to investigate the Mode-I fracture behavior of multi-material FFF interfaces as shown in Figure 1b. CT tests have been widely used to study Mode-I translaminar fracture of composites [31], as they allow a stable crack propagation. These tests were initially developed for metallic materials under ASTM-E399 [32], but they have since been adapted for composite materials [31] and polymers [33]. Accordingly, the ASTM E399 CT specimen geometry and calculation framework were used for the present CT configuration. Because the specimens were fabricated from dissimilar polymeric materials, the calculated (K_IC_) values are reported as apparent, geometry-normalized fracture parameters. The principal limitations include material anisotropy, nonlinear deformation, interface heterogeneity, and the absence of a demonstrated plane-strain validity check. The three-dimensional CAD model of the CT specimen was first created in SOLIDWORKS 2025 (Dassault Systèmes SolidWorks Corp., Waltham, MA, USA) and then exported as an STL file. The STL geometry was subsequently imported into Bambu Studio, where the printing parameters and material regions were defined. The CT specimen had a characteristic width of (W = 20) mm. The initial crack length was selected as (a = 0.45W), corresponding to approximately 9 mm, while the specimen thickness, (B), was approximately one-half of the specimen width. The nominal 9-mm initial crack was formed by the printed notch. Then, pre-crack followed using a blade to sharpen the crack tip, because without this pre-cracking, the finite notch radius could have overestimated the calculated K_IC_. The upper and lower portions of each CT specimen were assigned to different thermoplastic materials so that the crack path could be positioned at or associated with the dissimilar-material interface. This configuration enabled the resistance of the multi-material interface to opening-mode crack propagation to be evaluated. Each of these specimens was made using a Bambu Lab H2D FFF 3D printer. The nominal diameter of the filament used was 1.75 mm for each of the three types of filaments. Additionally, the specimens were printed with 100% infill density to reduce the effect of internal porosity from partially filled specimens. Processing conditions specific to each material type were used during the manufacturing process. PLA was printed at a nozzle temperature of 220 °C, a bed temperature of 55 °C, and a print speed of 200 mm/s. TPU was processed at a nozzle temperature of 230 °C, a bed temperature of 55 °C, and a print speed of 130 mm/s.

Nozzle temperature, bed temperature, and printing speed used to print PETG were 245 °C, 55 °C, and 200 mm/s, respectively. The samples were printed at a layer height of 0.2 mm with a printer nozzle diameter of 0.4 mm with a flat (XY plane) build orientation. The above conditions were chosen based on the printing parameters used for the respective Bambu Lab filament types. Prior to extrusion, all filaments were dried to prevent moisture-related problems in the extrusion process. PLA was dried at 50 °C for 8 h, PETG was dried at 65 °C for 8 h, and TPU was dried at 70 °C for 8 h in the heating oven. After drying, the materials and fabricated samples were placed in sealed boxes that contained desiccant to keep the relative humidity below 20%. The above measures were taken to facilitate uniform extrusion.

### 2.3. Raster Orientation

In addition to material matching, this study investigated the influence of raster orientation, which describes the effect of the infill deposition structure on interfacial fracture properties. Aligned rectilinear and rectilinear deposition structures were selected with 100% infill density to control the impact of raster angle on fracture behavior. Three raster orientations were examined for aligned rectilinear: 0°, 45°, and 90°. Two raster configurations were examined for alternating rectilinear: 0°/90° and +45°/−45°. Therefore, five deposition configurations were examined as shown in Figure 1a. These orientations have been chosen to study the effect of filament orientation with respect to the direction of loading and crack propagation on Mode-I fracture properties. Because FFF generates an anisotropic microstructure, any changes in raster orientation can affect the load transfer mechanism, stress distribution, and crack propagation resistance. Thus, a comparison of these five designs offers a systematic approach for analyzing the role of printing structure on interfacial fracture resistance of the multi-material specimens.

### 2.4. Mode-I Fracture Toughness Testing

The fracture-mechanics behavior of the multi-material samples was determined through compact-tension tests conducted in the Mode-I opening. The CT test was selected because it will create stress concentration at the tip of the crack and allow crack propagation through or along the multi-material interface. CT samples were subjected to tensile testing on a universal testing machine operating under controlled displacement conditions as shown in Figure 1c. Tensile loads were applied to the loading holes of the CT sample using clevis-type fixtures attached to a Lloyd Instruments Plus single column universal testing machine (UTM). Testing was performed at a constant crosshead displacement rate of 1 mm/min. The loading arrangement resulted in opening of the crack causing predominantly Mode-I fracture. The applied load and resulting displacement were recorded during each test to determine the peak load corresponding to crack propagation. Once the peak load was determined, the fracture toughness (*K_Ic_*) was calculated. The load and machine displacement were monitored until failure, and the maximum load was used for the calculation of Mode-I fracture parameters.

Using the peak load obtained from the experimental test, K_Ic_ can be determined using the Equation (1) [33]. The calculated stress-intensity factors were obtained using the compact-tension geometry and stress-intensity-factor formulation adopted from ASTM E399. The specimens had an (a/W) ratio of 0.45, which is appropriate for the selected CT geometry. The test specimens consisted of printed polymers with dissimilar material interfaces. The initial crack was formed on the printed notch before testing to compensate for printing fillet radius. This helps to control higher apparent K_IC_ values.

(1) $$K_{\mathrm{Ic}}=\frac{P_{\max}}{B\sqrt{W}}\;f\left(\frac{a}{W}\right)$$

where P_max_ is peak load, B is specimen thickness, W is specimen width, α is normalized crack length, and $f\left(\frac{\alpha }{W}\right)$ is the dimensionless geometry correction factor for the CT specimen.

The dimensionless geometry correction factor was calculated using Equation (2) [33].

(2) $$f\left(\alpha \right)=\frac{\left(2+\alpha \right)}{{\left(1-\alpha \right)}^{3/2}}\left(0.866+4.64\alpha -13.32\alpha^{2}+14.72\alpha^{3}-5.60\alpha^{4}\right)$$

The experimentally determined Mode-I fracture toughness, K_Ic_, was converted to the critical strain-energy release rate, G_Ic_, for implementation in the cohesive zone model. The conversion was performed using $G_{\mathrm{Ic}}\;=\;{K_{\mathrm{Ic}}}^{2}/E_{\mathrm{eff}}$ [34], where the effective elastic modulus of each dissimilar material pair was estimated as E_eff_ = 2E_1_E_2_/(E_1_ + E_2_). Young’s moduli of 3350, 2200, and 1190 MPa were adopted for PLA, PETG, and TPU, respectively, based on the material datasheet. The resulting G_Ic_ values were subsequently employed as Mode-I fracture energy governing damage evolution and crack propagation within the cohesive zone model. The effective moduli wereestimated based on Young’s moduli taken from the datasheets. These values are not direct measurements of the printed laminates and do not capture raster-dependent anisotropy, porosity, strain rate, or dissimilar-interface constraint. Consequently, the reported G_IC_ should be interpreted as an effective approximate estimate for comparative purposes rather than a fully validated interfacial material constant.

The estimated fracture toughness was utilized for the comparison of fracture resistance between different material pairs and raster directions. The higher the value is, the more resistant to Mode-I crack propagation; conversely, a low value is a sign of a high level of susceptibility to interface fracture. The comparison of fracture behavior between the PLA–TPU, PLA–PETG, and TPU–PETG materials was performed to identify the contribution of both the material pair and printing configuration to the achieved level of fracture resistance.

### 2.5. Cohesive Zone Modeling

**Figure 2:**
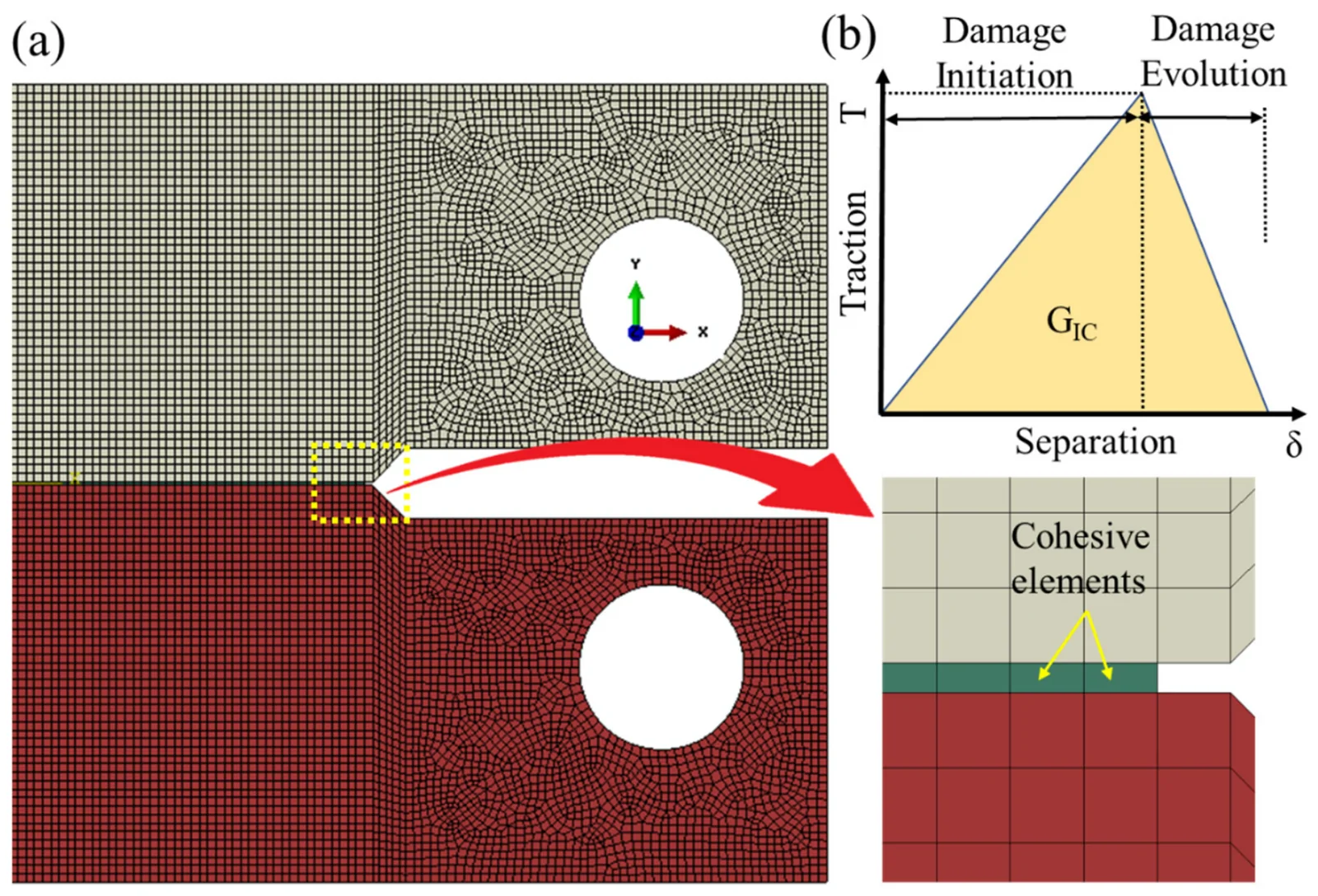
(**a**) FE model of the multi-material CT specimen showing mesh refinement and cohesive elements along the predefined fracture path. (**b**) Bilinear traction-separation law adopted for the CZM, where the enclosed area represents the Mode-I fracture energy, G_Ic_.

A cohesive zone modeling (CZM) approach was employed to simulate Mode-I crack initiation and subsequent damage along the predefined interface of the multi-material CT specimens. Finite-element simulations were performed using Abaqus/CAE 2023 with the Abaqus/Explicit solver (Dassault Systèmes Simulia Corp., Johnston, RI, USA). The bulk materials were modeled using an isotropic linear-elastic constitutive model. Young’s moduli of 3350 MPa for PLA, 2200 MPa for PETG, and 1190 MPa for TPU were used, with a Poisson’s ratio of 0.35 for all materials. As illustrated in Figure 2a, the fracture path was discretized using a thin layer of cohesive elements located between the two constituent materials, with local mesh refinement introduced around the crack tip and loading-hole region. The cohesive layer thickness was 0.1 mm, while a global element size of 0.2 mm was adopted for the surrounding bulk materials. The finite-element model consisted of COH2D4 four-node two-dimensional cohesive elements, CPS4R four-node bilinear plane-stress elements with reduced integration, and CPS3 three-node linear plane-stress triangular elements. To reproduce Mode-I loading, oppositely directed vertical displacements were applied to the upper and lower loading holes. The upper loading hole was displaced upward, while the lower loading hole was displaced downward, thereby opening the predefined interface.

The interfacial response was represented by a bilinear traction-separation law, as schematically shown in Figure 2b. Initially, the interface responds elastically until the maximum cohesive traction is reached, after which progressive stiffness degradation occurs until complete separation. Damage initiation was defined using the quadratic nominal-stress criterion, while damage evolution followed a linear softening law. The area under the traction-separation law represents the critical Mode-I fracture energy, G_Ic_, thereby providing an energy-based description of interfacial failure. The maximum cohesive traction was not measured directly as a local interfacial stress. Instead, it was calibrated separately for each of the 15 material-interface and raster-orientation combinations using an inverse procedure. The mean experimental maximum load was used as the target response, and maximum cohesive traction was iteratively varied until the predicted maximum load closely matched the corresponding experimental value. The experimentally derived G_Ic_ values were assigned as cohesive fracture energies, while a constant normal penalty stiffness of 2600 MPa/mm was used for all cases to ensure consistent interfacial stiffness during the comparative analysis. This penalty stiffness was treated as a numerical parameter and was not calibrated against the experimental maximum load.

**Figure A1:**
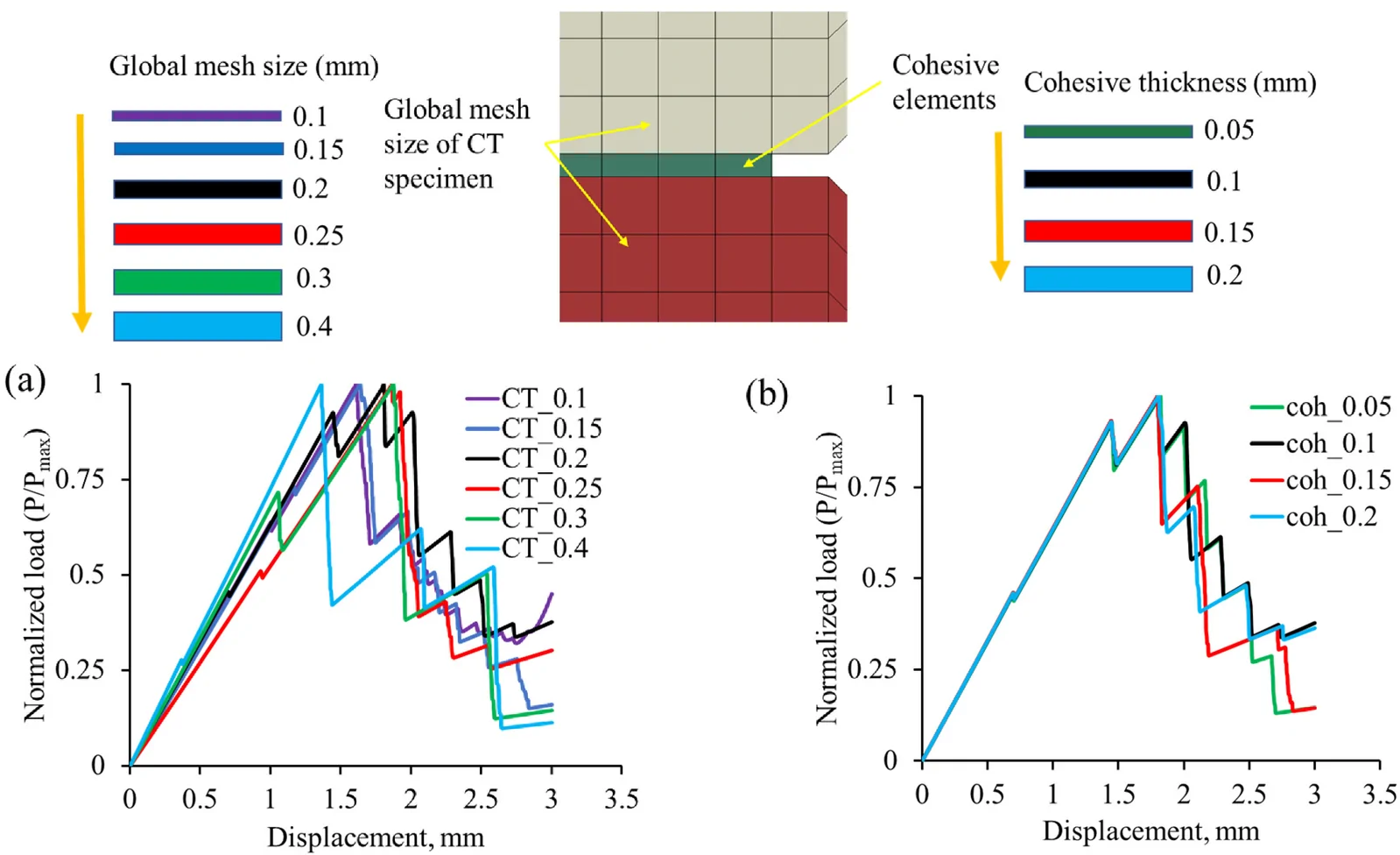
Mesh-sensitivity analysis using peak-normalized load-displacement responses (P/P_max_). (**a**) Effect of global bulk mesh size; and (**b**) effect of cohesive-layer thickness.

The bilinear law was selected as a simple yet effective representation of the predominantly brittle interfacial fracture observed in the multi-material specimens. Although the CZM formulation can simulate both damage initiation and subsequent crack propagation, the present analysis focused primarily on predicting fracture initiation and the corresponding maximum load. Mesh sensitivity was evaluated using peak-normalized load-displacement responses, and the results are provided in Appendix A (Figure A1). Figure A1a presents the global mesh-size study, in which the bulk mesh size varied between 0.1, 0.15, 0.2, 0.25, 0.3, and 0.4 mm while maintaining the cohesive-layer thickness at 0.1 mm. Figure A1b presents the cohesive-layer-thickness study, in which the cohesive-layer thickness was varied between 0.05, 0.1, 0.15, and 0.2 mm while maintaining the global bulk mesh size at 0.2 mm. In both figures, the load was normalized by the corresponding peak load, (P/P_max_). The selected global mesh size of 0.2 mm and cohesive-layer thickness of 0.1 mm produced a stable loading and post-peak softening response compared with the finer discretization. Accordingly, these values were used for all subsequent finite-element simulations.

### 2.6. Statistical Analysis

Two-way analysis of variance (ANOVA) was used to evaluate the effects of material pairs, raster orientation, and their interaction on peak load (P_max_). The analysis used the individual peak-load measurements from three replicate specimens for each material-pair and raster-orientation combination (n = 3). Statistical significance was assessed at a 5% significance level (*α* = 0.05). The material pair and raster orientation were treated as fixed factors, and the contribution of each source of variation was calculated from the analysis of variance table. Because K_IC_ was calculated from P_max_ using a common CT-specimen geometry, and G_IC_ was subsequently calculated from K_IC_, the statistical analysis was performed using P_max_ as the primary response variable. K_IC_ and G_IC_ are therefore presented as derived fracture parameters, and their differences are interpreted descriptively.

## 3. Results and Discussions

### 3.1. Load-Extension Response of Compact Tension Specimens

**Figure 3:**
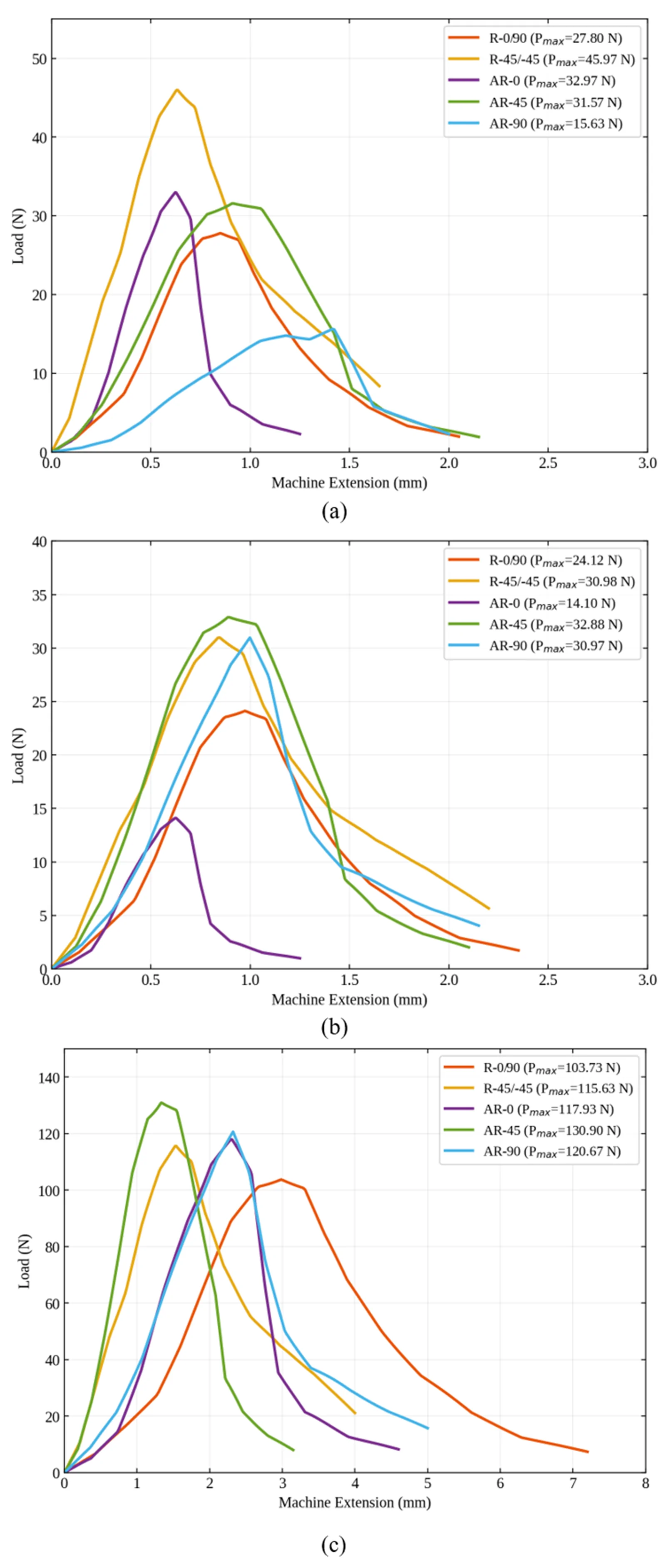
Load-machine extension curves of CT specimens for (**a**) PLA–PETG, (**b**) PLA–TPU, and (**c**) PETG–TPU interfaces at different raster orientations.

The load-machine extension plots shown in Figure 3 demonstrate the interfacial fracture behavior of CT specimens using three different material pairings of (a) PLA–PETG, (b) PLA–TPU, and (c) PETG–TPU. These different combinations of materials are tested at five different raster orientations under rectilinear (R) and aligned rectilinear (AR) (R-0/90, R-45/−45, AR-0, AR-45, and AR-90). The curves show descriptive differences in maximum load, deformation to failure, and post-peak behavior. This shows that there are different fracture toughness and energy dissipation mechanisms at the interface. Overall, the load was gradually increasing with machine extension until it reached a peak value, after which all specimens show varying levels of post-peak softening related to crack propagation and the loss of load carrying capacity. For most of the curves there is a sudden jump to the maximum value followed by progressive softening. The most striking contrast lies in the intrinsic material differences.

For the rigid–rigid PLA–PETG system (Figure 3a), the mechanical response is characterized by moderate average peak loads ranging from 15.63 N to 45.97 N. It was accompanied by steep initial elastic slopes and rapid post-peak load drops within an extension window of 1.25 mm to 2.2 mm, indicative of brittle interfacial debonding. On the other hand, the rigged-flexible PLA–TPU interface (Figure 3b) is the weakest interface in terms of load-carrying capacity with the average peak loads ranging from 14.10 N to 32.88 N but relatively broader curves, suggesting moderate ductility. In contrast, the PETG–TPU interface (Figure 3c) shows a significant improvement in fracture strength, attaining average peak loads ranging from 103.73 N to 130.90 N. It is about a three-to-four-fold improvement compared to the PLA-based interface systems. The machine extensions extend up to 7.5 mm, indicating a highly ductile and tough interface between these material pairs.

Regarding raster orientation, there is no clear trend and pattern on the curves as material pairs. Relatively AR-45 and R-45/−45 infill patterns generally yield higher average peak loads across the material systems. Descriptively, the PLA–PETG R-45/−45 specimens showed the highest mean peak load, whereas PLA–TPU AR-90 showed the lowest mean peak load. On the other hand, AR-0 shows a sudden drop in the post-peak region. Overall, the fracture performance of FFF parts made of multi-materials is more influenced by the pairing of materials than raster orientation. The PETG–TPU combination had the maximum overall average peak-load fracture resistance and extension, whereas the interfaces with PLA were lower.

### 3.2. Effect of Material Pairing and Raster Orientation on Peak Load

**Figure 4:**
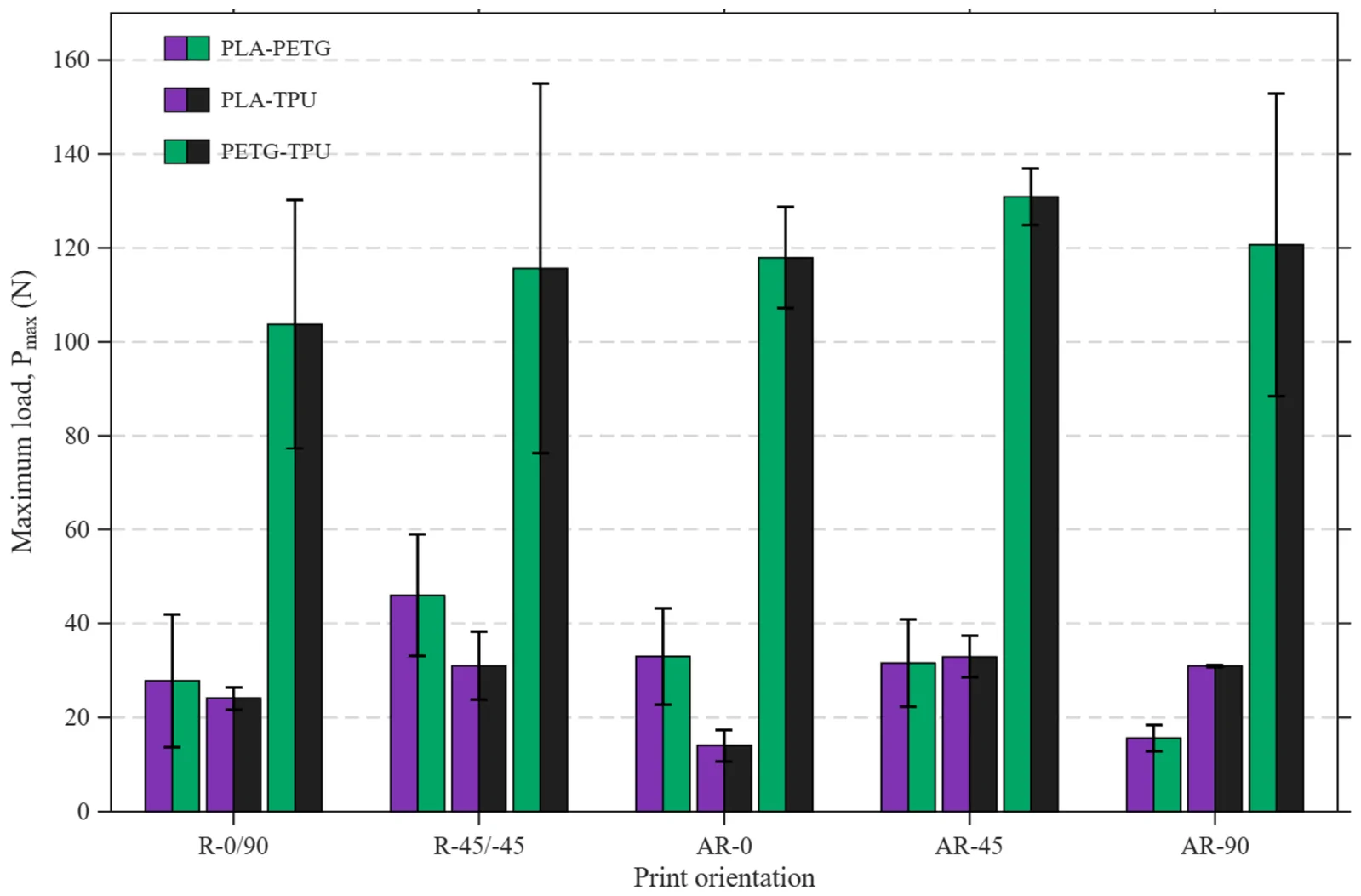
Effect of material interface pairing and raster-print orientation on the peak load of CT specimens.

The bar chart presented in Figure 4 shows the average peak load (P_max_) of multi-material FFF CT specimens for five different printing orientations (R-0/90, R-45/−45, AR-0, AR-45, and AR-90). Three different interfaces are compared, PLA–PETG (purple/green), PLA–TPU (purple/black), and PETG–TPU (green/black). The error bars show the standard deviation. From the results, it is evident that the material pairs have a significant effect on the load-carrying ability of the interfaces than raster orientation. Notable performance disparity exists in material pairs where the PETG–TPU pair proves to be superior to both PLA-based pairs across every raster orientation. It attains an average peak load of 103.73 N–130.90 N. This showed there is a high interface between PETG and TPU. On the other hand, the average peak load for the PLA–PETG and PLA–TPU pairs remains constrained less than 46 N across all raster orientations. These results together with the ANOVA clearly identify material pairing as the dominant factor controlling the load-carrying capacity of the multi-material FFF interfaces.

AS PETG–TPU exhibited better performance in terms of peak load compared to PLA-based blends, PETG–TPU demonstrated a higher resistance to Mode-I interfacial debonding at the applied printing conditions, along with the toughness of PETG and the ductility of TPU, making it possible to achieve better stress transfer and redistribution of crack tip stress. The flexibility of TPU can be used to cause stress redistribution and crack-tip blunting, while the interaction of PETG and TPU might lead to formation of the more mechanically resistant interface than PLA-containing blends. The enhanced fracture resistance of the PETG–TPU interface may be attributed to the combined effects of interfacial wetting, thermally assisted polymer-chain diffusion, physical interactions, TPU-induced ductile deformation, and mechanical interlocking. Previous studies have shown that interfacial bonding in multi-material FFF is influenced by thermal history and intermolecular diffusion [35]. In addition, PETG–TPU blends have been reported to exhibit predominantly physical mixing without evidence of a new chemical reaction [36].

Print-raster orientation determines the alignment of the polymer strands relative to the direction of the applied load, which is a hallmark of AM anisotropy. Here there is no significant change on the average peak load as the raster orientation changes. The patterns are changed as the material pair changes. Descriptively, PLA–PETG had its highest mean peak load for R-45/−45 (45.97 N) and its lowest for AR-90 (15.63 N). PLA–TPU had its highest mean for AR-45 (32.88 N) and its lowest for AR-0 (14.10 N). PETG–TPU had its highest mean for AR-45 (130.90 N) and its lowest for R-0/90 (103.73 N). These within-pair rankings are descriptive because the main effect of raster orientation was not statistically significant and the interaction with material pair was also not significant. The measured data contains configuration-level differences, but the two-way ANOVA indicates that the effect of raster orientation was not statistically significant. PETG–TPU/AR-45 produced the highest observed peak-load mean in the tested dataset, while the material-pair effect accounted for most of the explained variation. Thus, PETG–TPU can be identified as the material pair with the highest average response under the investigated condition. The peak-load results were therefore interpreted together with the fracture-mechanics parameters below.

Two-way analysis of variance (ANOVA) was performed with a 5% significance level as shown in Table 1. The results showed that material pairs had a significant effect on the measured response (peak load) with *p* < 0.0001. This demonstrates that the average response differed considerably among PLA–TPU, PLA–PETG, and PETG–TPU. Material pairs proved to be the most influential factor in the effect on the peak load, accounting for 83.66% of the total variation. On the other hand, print orientation showed no statistically significant impact, *p* = 0.5998, implying that the effect from the five different orientations was relatively insignificant compared to the experimental result variability. The print orientation accounted for just 1.21% of the total variation, hence indicating that the different orientations had minimal effect on the response. The interaction effect of the material pair and print orientation accounted for 2.16%, which was insignificant, *p* = 0.7520. It implies that the material pairs had similar effects under different orientations. Residual error had accounted for 12.98% of the total variation in the peak load response. Consequently, the variation in response was largely due to the material combination and not the orientation of printing or their interaction.

Thus, material pairs showed the significant effect. PETG–TPU exhibited higher mean peak loads than PLA–TPU and PLA–PETG across the tested configurations, as shown in Figure 4. The orientation effects were comparatively small relative to the residual variability and were not statistically significant. In general, PLA–TPU with AR-0 raster orientation had the lowest observed average peak load. PETG–TPU with AR-45 raster orientation had the highest observed average peak load. These individual rankings should be interpreted cautiously because the analysis does not support a raster-orientation effect. These results suggest that PETG–TPU may provide comparatively stable performance across the printing orientations investigated, although considerable variability was observed for some configurations.

### 3.3. Mode-I Fracture Toughness (K_IC_) and Critical-Strain Energy-Release Rate (G_IC_)

The fracture toughness (K_IC_) of the multi-material FFF interfaces was also assessed in terms of the Mode-I critical-stress intensity factor (Fracture Toughness), and critical-strain energy-release rate, G_IC_. In contrast to peak load, K_IC_ considers the CT specimen geometry and describes the critical value of crack-tip stress intensity, while G_IC_ gives the energy criterion of resistance to crack propagation in Mode I. Given the specific CT geometry used in the present investigation, a/W = 0.45 and f (a/W) = 8.22. Similarly, the G_IC_ value was calculated from the resulting K_IC_. For each material pair and raster orientation, three replicate CT specimens were tested (n = 3) (i.e., three material pairs × five raster orientations × three replicates = forty − five total specimens). The average values along with the standard deviation are reported in Table 2.

**Figure 5:**
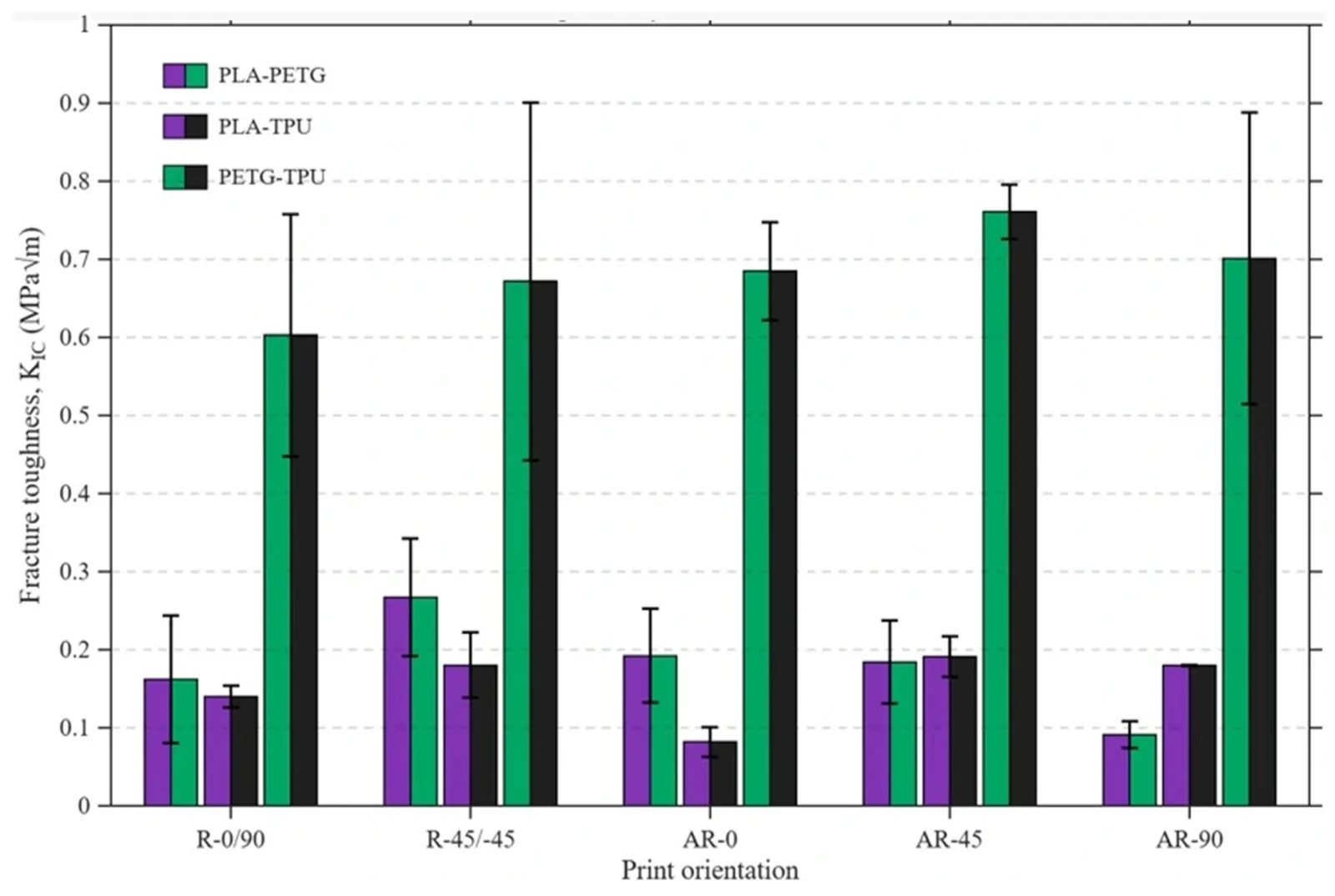
Fracture toughness (K_IC_) of CT results under different material composition and raster-print orientation.

The results demonstrate that there is a significance difference in fracture behavior between CT samples with material pairs under multi-material FFF. Figure 5 illustrates the K_IC_ values computed from the CT test for all three material pairs printed at five raster-print orientations (R-0/90, R-45/−45, AR-0, AR-45, and AR-90). Three different interfaces are PLA–PETG (purple/green), PLA–TPU (purple/black), and PETG–TPU (green/black). The bar chart clearly demonstrates that material pairing significantly affects interfacial fracture resistance. Results show an extremely high level of interfacial polymer pair sensitivity, where the PETG–TPU combination is far superior compared to both PLA combinations for any raster orientation. The combination of PETG and TPU showed the highest K_IC_ value at any raster direction from 0.603 $\mathrm{MPa}\sqrt{m}$ to 0.761 $\mathrm{MPa}\sqrt{m}$. This is roughly a three- to four-fold improvement in crack resistance compared to PLA–PETG and PLA–TPU, which is below 0.27 $\mathrm{MPa}\sqrt{m}$. It should be noted that the maximum values of K_IC_ for PLA–PETG and PLA–TPU were 0.267 and 0.191 $\mathrm{MPa}\sqrt{m}$, respectively. This disparity highlights the critical role of material pair and interlayer adhesion in multi-material FFF. Even though raster orientation governs the local stress state and crack path tortuosity across material pairs, its effect is material dependent. The K_IC_ results are consistent with P_max_ trends in Figure 3; material pairs with higher peak loads generally also show higher fracture toughness. Overall, PETG–TPU offers the best combination of load-bearing capacity and fracture toughness.

In this study the energy required to propagate a crack per unit area of interfacial fracture was investigated using the critical-strain energy release rate (G_IC_). It accounts for both the stress intensity and the elastic modulus of the material system, considered a more direct measure of fracture resistance. The results in Table 2 show that G_IC_ is strongly dependent on material pairings and the trends closely mirror those observed for *P*_max_ and *K_IC_*. The most dominant factor is the material pair. The results show that there is a great difference in energy absorption capacity during Mode-I crack propagation between the materials. The PETG–TPU combination is superior to the PLA-based interface, where G_IC_ value lies between 250.8 and 375.5 N/m. On the other hand, the G_IC_ value for PLA–PETG varies between 3.2 N/m and 29.0 N/m, while PLA–TPU varies between 4.0 N/m and 21.2 N/m.

The elevated values of K_IC_ and G_IC_ for PETG–TPU illustrate the considerable increase of the resistance against crack initiation and propagation. Deformability of TPU could have contributed to the blunting of cracks, stress redistribution, and energy dissipation. But it is clear from the findings that the properties of TPU alone do not have effect on the fracture resistance because PLA–TPU showed significantly lower values. It is observed from the findings that the combination of materials and their interface properties dominate the process of fracture resistance. The influence of raster orientation was not significant as material pairs. It has less of an effect on the response compared to the material pair. Its effect varies as the material pair varies. For example, for PLA–PETG, the highest and lowest values of fracture resistance (K_IC_ and G_IC_) were found at R-45/−45 and AR-90 raster orientations respectively. For the PLA–TPU pair, the maximum K_IC_ and G_IC_ was achieved by AR-45 raster orientation, whereas AR-0 raster orientation had minimum values. For the PETG–TPU pair, the highest fracture resistance was provided by AR-45 orientation and the lowest was found at R-0/90 raster orientation.

Overall, interfacial fracture performance is mainly controlled by material pairing. PETG–TPU consistently gives the highest *P*_max_, *K_IC_*, and *G_IC_*, compared to PLA–PETG and then PLA–TPU. Among all tested compositions, the PETG–TPU with AR-45 showed the highest general fracture resistance to Mode I, which corresponds to P_max_ of 130.90 N, K_IC_ of 0.761 $\mathrm{MPa}\sqrt{m}$, and G_IC_ of 375.5 N/m. Raster orientation was not significant and it varies with material pair. Thus, the selection of materials is the decisive factor for the general fracture resistance level while manufacturing multi-material FFF parts.

### 3.4. Fractured-Sample Characterization

**Figure 6:**
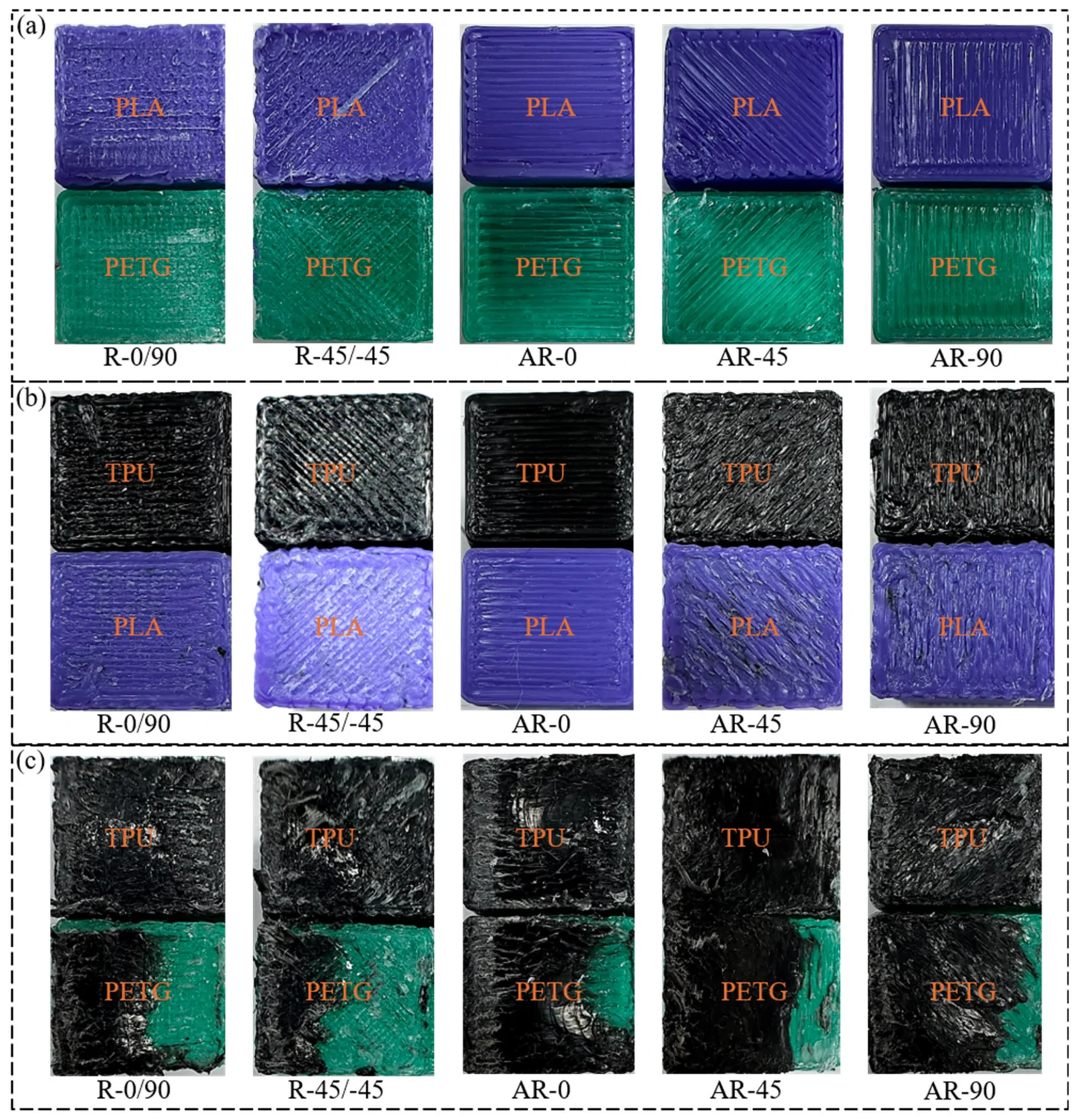
Fractured surfaces of (**a**) PLA–PETG, (**b**) PLA–TPU, and (**c**) PETG–TPU interfaces for R-0/90, R-45/−45, AR-0, AR-45, and AR-90 raster orientations, showing both mating surfaces after CT test of Mode-I fracture.

Figure 6 shows the fracture surfaces of PLA–PETG (see Figure 6a), PLA–TPU (see Figure 6b), and PETG–TPU (see Figure 6c) for all five rasters. Both sides of the fractured interfaces were evaluated in terms of material transfer, surface morphology, and effect of raster geometry on the fracture process. The fracture surfaces differ for each of the three materials combinations, reflecting differences in adhesion and interlocking at the interface. In the case of PLA–PETG, the original raster structures can be distinctly seen from both sides of the fractured samples. The fracture happened at the interface due to the relatively regular structure of filament lines without observing material transfer. Moreover, the surface morphology changes depending on the orientation of the rasters, matching the directions of deposited filaments. The inclined raster configurations (R-45/−45 and AR-45) resulted in more complex fracture-surface geometries compared to the 0° and 90° rasters. For the case of the PLA–TPU fracture surfaces, the degree of surface deformation and roughness appears to be higher especially for the inclined and transverse rasters. Irregularities and traces left on the opposite surfaces are a manifestation of stronger contact between the deposited tracks during the fracture process. The smoother appearance of the AR-0 indicates its lower fracture resistance in comparison with other samples such as AR-45, R-45/−45, and AR-90. The most significant morphological features can be identified for the case of PETG–TPU. There is evidence of considerable material transport on both mating fracture surfaces, and the raster pattern is less visible compared to those of PLA–PETG and PLA–TPU. The rough nature of the surface implies that crack growth was associated with considerable deformation and mechanical interlocking as opposed to separation along a clean surface. Such an inference agrees with the significantly larger P_max_, K_IC_, and G_IC_ recorded in PETG–TPU. Notably, all raster orientation specimens had evidence of extensive deformation and material transport because it had the highest Mode-I fracture resistance.

In terms of FFF bonding, the adhesion at the interface is achieved by means of intimate contact and polymer-chain diffusion. Interfacial adhesion is promoted by intimate contact and polymer-chain diffusion while the deposited materials remain sufficiently warm, as well as by mechanical interlocking caused by the geometry of the deposited roads. The fracture images alone cannot quantify the molecular diffusion, but the higher degree of material transfer and more uneven morphology of the fracture for PETG–TPU is indicative of a stronger interface due to increased interaction between the materials. In general, the fracture surface observations corroborate the mechanical test results showing higher roughness, material transfer, and complexity in the path of fracture. The fracture-surface observations in this study were qualitative. Consequently, the descriptions of surface roughness, material transfer, crack deflection, and mechanical interlocking should be considered interpretations based on the observed fracture morphology rather than quantitatively verified mechanisms. Quantitative measurements are recommended for future validation.

### 3.5. Comparison Between Experimental and FE Simulation Results

To assess the consistency of the cohesive-zone model with the experimentally observed fracture-initiation response and maximum-load response, the finite-element results were compared with the experimental findings for all material interfaces and raster orientations. Because the maximum cohesive-traction values were calibrated using the mean experimental maximum load for each material-interface and raster-orientation combination, this comparison represents an internal calibration assessment rather than independent validation. Accordingly, the agreement with the experimental load response should be interpreted as reproduction of the calibration dataset rather than as an independent prediction.

**Figure 7:**
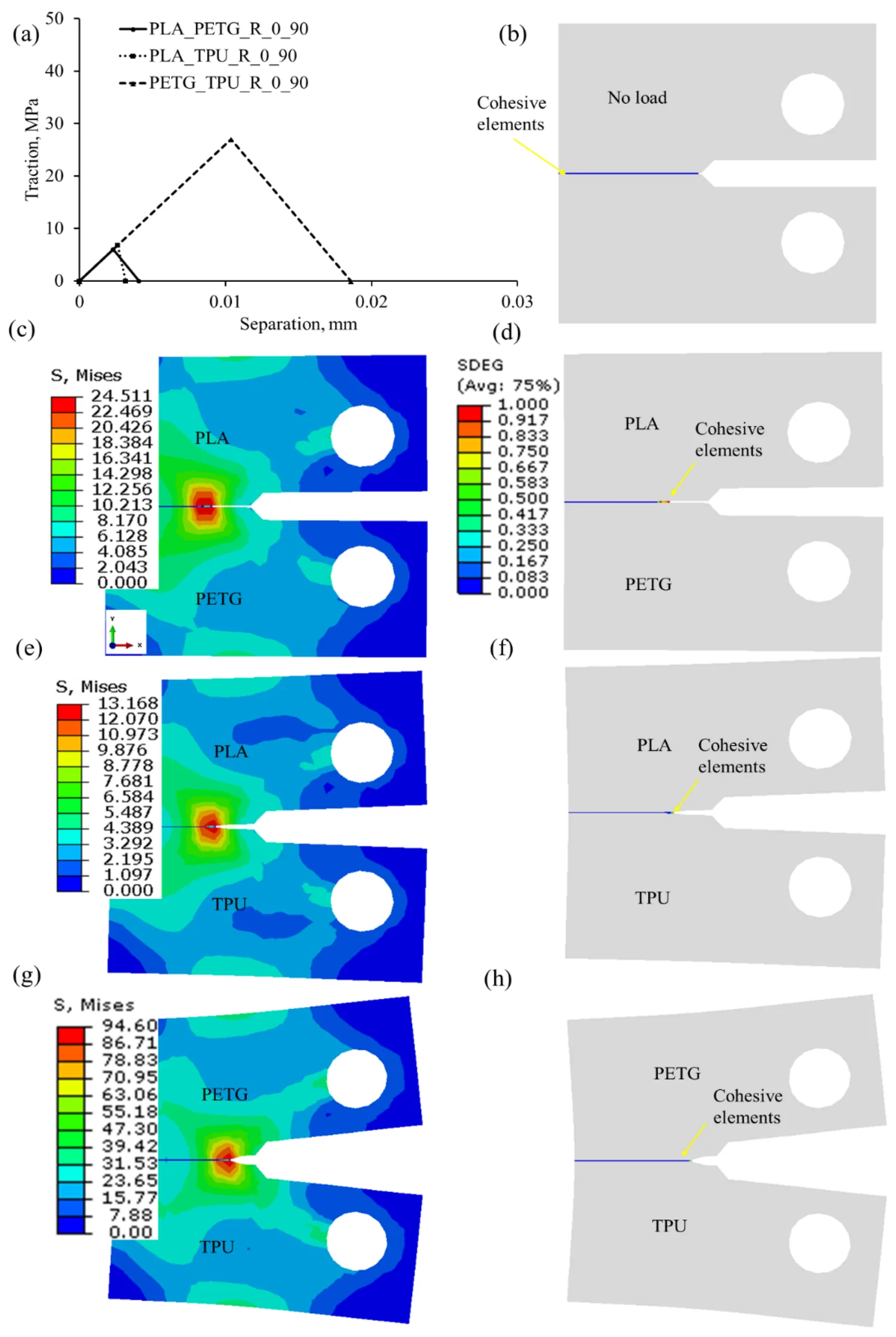
Finite-element results for the R-0/90 configuration: (**a**) traction-separation response, (**b**) unloaded configuration, (**c**,**e**,**g**) von Mises stress distributions, and (**d**,**f**,**h**) cohesive-element damage for the PLA–PETG, PLA–TPU, and PETG–TPU interfaces, respectively.

Figure 7 shows the finite-element response of the R-0/90 specimens. The traction-separation curve in Figure 7a reaches the prescribed maximum cohesive traction before softening, indicating the onset of interfacial damage. The von Mises stress contours in Figure 7c,e,g reveal pronounced stress concentrations around the crack tip for the PLA–PETG, PLA–TPU, and PETG–TPU interfaces, respectively. The corresponding cohesive-element damage contours in Figure 7d,f,h confirm that damage is localized near the crack tip. Although the stress and damage patterns are qualitatively similar for all three interfaces, their magnitudes vary according to the respective cohesive strengths and material stiffnesses. These results show that the calibrated model reproduces the localized interfacial damage initiation and the associated maximum-load response for the R-0/90 configuration.

**Figure 8:**
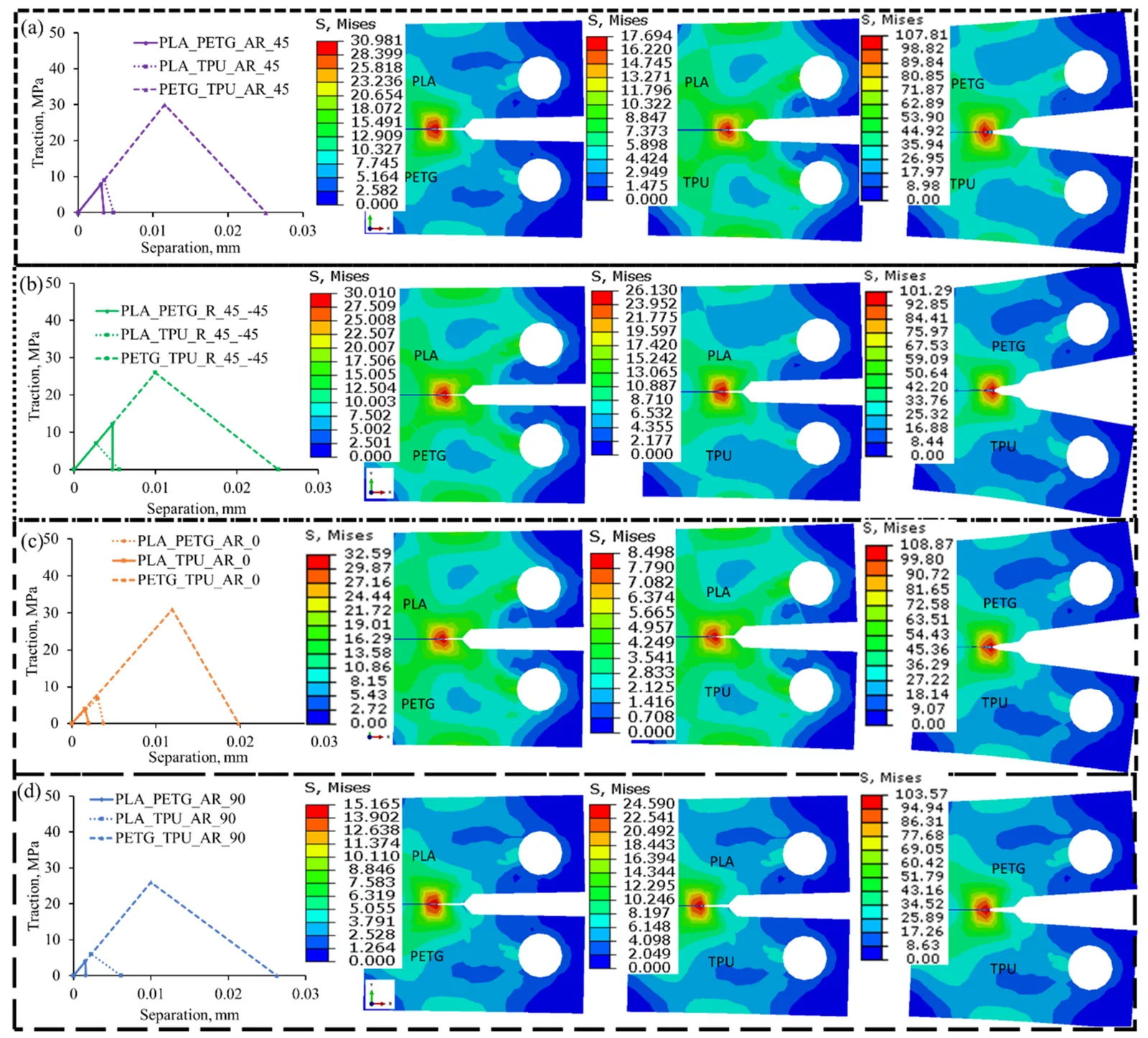
Finite-element results for the PLA–PETG, PLA–TPU, and PETG–TPU interfaces showing (**a**) R-45/−45, (**b**) AR-0, (**c**) AR-45, and (**d**) AR-90 printing orientations, together with the traction-separation responses and corresponding von Mises stress distributions.

Similarly, Figure 8 presents the FE results for the remaining printing orientations. Figure 8a–d correspond to the R-45/−45, AR-0, AR-45, and AR-90 configurations, respectively. Each row includes the traction-separation response and the von Mises stress distributions for the PLA–PETG, PLA–TPU, and PETG–TPU interfaces. The curves exhibit an increase in cohesive traction up to fracture initiation followed by progressive softening. In all cases, the highest stresses are concentrated near the crack tip, although their magnitude and distribution vary with the interface type and printing orientation. Because the experimental results and *ANOVA* indicated that raster orientation did not have a statistically significant effect on the maximum load, with (*p* = 0.5998), and the material-pair-raster-orientation interaction was also not statistically significant, with (*p* = 0.7520), the individual raster roads were not explicitly modeled in the finite-element analysis. Instead, each bulk material region was represented as a homogeneous and isotropic continuum. Therefore, the raster labels in Figure 7 and Figure 8 identify the corresponding experimental configurations rather than separate raster-resolved material models. The qualitatively similar stress distributions and cohesive-damage patterns across the configurations are consistent with the statistically nonsignificant effect of raster orientation.

Although raster architecture was not explicitly represented, the corresponding calibrated cohesive parameters were used for the respective material-interface and raster-orientation combinations. Thus, any differences between the cases should be interpreted as differences in the calibrated interfacial response rather than as a direct mechanistic prediction of raster-dependent bulk anisotropy.

Table 3 compares the finite-element predicted maximum loads with the corresponding average-experimental maximum loads. The predicted maximum loads generally showed good agreement with the average experimental maximum loads. However, because the cohesive-zone parameters were calibrated using the same experimental maximum load, the differences reported in Table 3 represent the agreement of the calibrated model with the calibration data rather than errors from an independent prediction. The stress and damage contours therefore provide qualitative evidence of localized interfacial damage within the calibrated model. To quantify this agreement, relative deviations were calculated by comparing the finite-element predicted maximum loads with the corresponding average experimental maximum loads.

For the PLA–PETG interface, the relative deviations ranged from −22.74% to 12.24%, with the largest deviation observed for the R-45/−45 orientation. The PLA–TPU interface showed relative deviations between −10.27% and 8.97%. The PETG–TPU interface exhibited the closest agreement, with relative deviations ranging from −3.61% to 3.80%. Overall, these comparisons demonstrate that the calibrated cohesive-zone parameter sets reproduce the experimentally observed load-carrying response and localized interfacial damage patterns. A separate experimental dataset that was not used to calibrate the maximum cohesive traction parameter would be required for independent validation. This is beyond the scope of the present study and will be addressed in future work.

## 4. Conclusions

This study investigated the Mode-I fracture behavior of PLA–PETG, PLA–TPU, and PETG–TPU interfaces fabricated by multi-material FFF using CT specimens with different raster configurations. Both rectilinear (R) and aligned rectilinear (AR) configurations were examined, including R-0/90, R-45/−45, AR-0, AR-45, and AR-90. The results demonstrated that material pairing had a statistically significant and dominant effect on peak load, whereas raster orientation and its interaction with material pair were not statistically significant in the reported analysis of variance (ANOVA). Among the investigated combinations, the PETG–TPU interface exhibited the highest peak load (P_max_), Mode-I fracture toughness (K_IC_), and critical-strain energy-release rate (G_IC_). In contrast, the PLA-based interfaces showed lower fracture resistance.

Fracture-surface observations supported these findings, revealing greater deformation, material transfer, surface roughness, and mechanical interlocking in the PETG–TPU specimens. However, the fracture-surface observations were qualitative. Therefore, the descriptions of surface roughness, material transfer, crack deflection, and mechanical interlocking should be interpreted as observations based on the fracture morphology rather than as quantitatively verified mechanisms.

A cohesive-zone model incorporating quadratic nominal-stress damage initiation and linear damage evolution was used to simulate the interfacial response. The calibrated model reproduced the localized cohesive damage initiation and the corresponding maximum-load response near the crack tip. Because the maximum cohesive traction values were calibrated using the experimental maximum-load data, the finite-element comparison represents an internal calibration assessment rather than independent model validation.

Overall, the combined experimental and numerical results indicate that material pairing is the primary factor governing the Mode-I fracture resistance of the investigated multi-material FFF interfaces. Raster orientation had no statistically significant effect under the tested conditions and was therefore modeled using homogeneous and isotropic bulk material domains without explicitly resolving individual raster roads. The influence of raster-dependent behavior should be further investigated using independent experiments and raster-resolved or anisotropic finite-element models.

## Figures and Tables

**Table 1:** Two-way ANOVA of material pair and orientation.

Source	Sum of Square (*SS*)	Degrees of Freedom (*DF*)	Mean Square (*MS*) = $\left(\frac{\mathrm{SS}}{\mathrm{DF}}\right)$	F-Statistic $F=\left(\frac{{\left(\mathrm{MS}\right)}_{\mathrm{source}}}{{\left(\mathrm{MS}\right)}_{\mathrm{error}}}\right)$	*p*	Contribution (%) $\left(\frac{{\left(\mathrm{SS}\right)}_{\mathrm{source}}}{{\left(\mathrm{SS}\right)}_{\mathrm{total}}}\times 100\right)$
Material Pair	79,697.39	2	39,848.70	96.67	<0.0001	83.66
Raster Orientation	1149.74	4	287.43	0.70	0.5998	1.21
Material Pair × Orientation	2053.61	8	256.70	0.62	0.7520	2.16
Error (Residual)	12,366.29	30	412.21	—	—	12.98
Total	95,267.03	44	—	—	—	100.00

**Table 2:** Effect of material pairing and raster-print orientation on peak load (P_max_), Mode-I fracture toughness (K_IC_), and critical-strain energy-release rate (G_IC_).

Orientation	Interface	P_max_ ± SD (N)	K_IC_ ± SD $\left(\mathrm{MPa}\sqrt{m}\right)$	G_IC_ ± SD (N/m)
R-0/90	PLA–PETG	27.80 ± 14.04	0.162 ± 0.082	12.3 ± 10.5
	PLA–TPU	24.12 ± 2.36	0.140 ± 0.014	11.3 ± 2.2
	PETG–TPU	103.73 ± 26.59	0.603 ± 0.155	250.8 ± 114.2
R-45/−45	PLA–PETG	45.97 ± 12.87	0.267 ± 0.075	29.0 ± 14.0
	PLA–TPU	30.98 ± 7.27	0.180 ± 0.042	19.5 ± 7.6
	PETG–TPU	115.63 ± 39.40	0.672 ± 0.229	326.4 ± 195.0
AR-0	PLA–PETG	32.97 ± 10.27	0.192 ± 0.060	15.2 ± 8.8
	PLA–TPU	14.10 ± 3.33	0.082 ± 0.019	4.0 ± 2.0
	PETG–TPU	117.93 ± 10.81	0.685 ± 0.063	306.7 ± 57.6
AR-45	PLA–PETG	31.57 ± 9.17	0.184 ± 0.053	13.7 ± 8.0
	PLA–TPU	32.88 ± 4.41	0.191 ± 0.026	21.2 ± 5.9
	PETG–TPU	130.90 ± 6.04	0.761 ± 0.035	375.5 ± 34.9
AR-90	PLA–PETG	15.63 ± 2.86	0.091 ± 0.017	3.2 ± 1.1
	PLA–TPU	30.97 ± 0.24	0.180 ± 0.001	18.4 ± 0.3
	PETG–TPU	120.67 ± 32.21	0.701 ± 0.187	341.1 ± 168.5

**Table 3:** Cohesive-zone parameters and comparison between the experimental and numerical maximum loads for the investigated material interfaces and printing orientations.

Interface	Orientation	Maximum Traction (MPa)	Normal Penalty Stiffness (MPa/mm)	Maximum Experimental Load (N) P_max_ ± SD	Maximum Numerical Load (N)	Error%
PLA–PETG	R-0/90	6	2600	27.80 ± 14.04	27.76	−0.13
	R-45/−45	12.25	2600	45.97 ± 12.87	35.52	−22.74
	AR-0	8	2600	32.97 ± 10.27	37.00	12.22
	AR-45	8	2600	31.57 ± 9.17	35.43	12.24
	AR-90	4	2600	15.63 ± 2.86	17.34	10.93
PLA–TPU	R-0/90	6.8	2600	24.12 ± 2.36	22.44	−6.96
	R-45/−45	7	2600	30.98 ± 7.27	29.85	−3.64
	AR-0	4	2600	14.10 ± 3.33	12.65	−10.27
	AR-45	9	2600	32.88 ± 4.41	35.83	8.97
	AR-90	6	2600	30.97 ± 0.24	28.29	−8.66
PETG–TPU	R-0/90	27	2600	103.73 ± 26.59	106.55	2.75
	R-45/−45	26	2600	115.63 ± 39.40	113.64	−1.70
	AR-0	31	2600	117.93 ± 10.81	122.38	3.80
	AR-45	30	2600	130.90 ± 6.04	133.86	2.26
	AR-90	26	2600	120.67 ± 32.21	116.25	−3.61

## Data Availability

The original contributions presented in this study are included in the article. Further inquiries can be directed to the corresponding authors.
